# AgriPest: A Large-Scale Domain-Specific Benchmark Dataset for Practical Agricultural Pest Detection in the Wild

**DOI:** 10.3390/s21051601

**Published:** 2021-02-25

**Authors:** Rujing Wang, Liu Liu, Chengjun Xie, Po Yang, Rui Li, Man Zhou

**Affiliations:** 1 Institute of Intelligent Machines, and Hefei Institute of Physical Science, Chinese Academy of Sciences, Hefei 230031, China; rjwang@iim.ac.cn (R.W.); cjxie@iim.ac.cn (C.X.); lirui@iim.ac.cn (R.L.); manman@mail.ustc.edu.cn (M.Z.); 2 Science Island Branch of Graduate School, University of Science and Technology of China, Hefei 230026, China; 3 Department of Computer Science, University of Sheffield, Sheffield S1 1DA, UK; po.yang@sheffield.ac.uk

**Keywords:** pest detection, agricultural dataset, AgriPest, deep learning

## Abstract

The recent explosion of large volume of standard dataset of annotated images has offered promising opportunities for deep learning techniques in effective and efficient object detection applications. However, due to a huge difference of quality between these standardized dataset and practical raw data, it is still a critical problem on how to maximize utilization of deep learning techniques in practical agriculture applications. Here, we introduce a domain-specific benchmark dataset, called AgriPest, in tiny wild pest recognition and detection, providing the researchers and communities with a standard large-scale dataset of practically wild pest images and annotations, as well as evaluation procedures. During the past seven years, AgriPest captures 49.7K images of four crops containing 14 species of pests by our designed image collection equipment in the field environment. All of the images are manually annotated by agricultural experts with up to 264.7K bounding boxes of locating pests. This paper also offers a detailed analysis of AgriPest where the validation set is split into four types of scenes that are common in practical pest monitoring applications. We explore and evaluate the performance of state-of-the-art deep learning techniques over AgriPest. We believe that the scale, accuracy, and diversity of AgriPest can offer great opportunities to researchers in computer vision as well as pest monitoring applications.

## 1. Introduction

Object detection is a classic research topic in the computer vision communities. The current large volume of standardized object detection datasets [1,2,3] help to explore many key research challenges that are related to object detection and evaluate the performance of different algorithms and technologies. Especially, the recent popularity and development of deep learning techniques has proved a fact that, given sufficient high-quality annotated image datasets, deep learning approaches [4,5,6] can effectively and efficiently achieve the detection and classification tasks. This results in some practical breakthroughs in many classic applications, including face recognition [7] and vehicle detection [8]. However, in some domain-specific object detection applications, there is a huge difference of quality between standardized annotated dataset and practical raw data. This leads us to the obvious question: how could we maximize the utilization of deep learning techniques in practical applications?

Taking an example of typical object detection in smart agriculture application, current pest monitoring task requires precise and pest detection and population counting in static image. In this case, computer vision based automatic pest monitoring techniques have been widely used in real practice. These computer vision techniques deal with pest images that were captured from fixed stationary, and then adopt traditional image processing algorithms to analyze the pest associated features for detection [9]. During this processing, most of the solutions aim to formulate it as a whole image classification task [10,11,12]. However, in practical applications, wild pest detection that requires not only classification, but also localization might be much more important for pest hazard assessment, since a precise detection performance could provide higher semantic information, such as pest occurrence areas and pest population counting information in the field.

**Figure 1:**
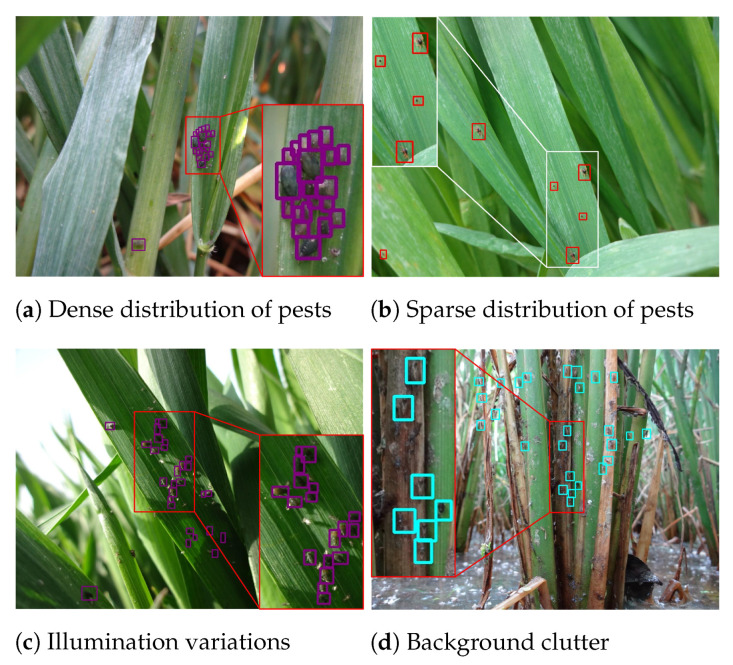
Example images of AgriPest. These samples indicate four types of typical scenes in pest detection task.

Despite recent deep learning approaches have showed great success in image recognition [13] or generic object detection applications [14,15,16], they are often intractable to be ready-to-use practical methods for showing satisfied performance on pest detection and classification. Towards this problem, the main reasons are: (1) when comparing with generic object detection, pest detection in the wild remains an open problem due to a challenging fact that many discriminative details and features of object are small, blurred, hidden, and short of sufficient details. These pose a fundamental dilemma that it is hard to distinguish small object from the generic clutter in the background. (2) The diversity and complexity of scenes in the wild cause a variety of challenges, including dense distribution, sparse distribution, illumination variations, and background clutter shown in Figure 1. These types of scenes might increase the difficulty of applying generic object detection techniques into tiny wild pest detection task.

It is well known that the large-scale image dataset plays a key role in driving efficient model and enables powerful feature representation. In the field of agricultural pest controlling, the first challenge is how to select the field crops and pest species in the large-scale dataset to build hierarchical taxonomy. From the practical point view of pest reduction, we consider the field crops that occupy a larger production of food in the world. Under this consideration, the Food and Agriculture Organization of the United Nations (FAO) reports that rice (paddy), maize (corn), and wheat are three major field crops for food production that could provide 700 M, 1000 M, and 800 M tones in 2019 [17]. Besides, there is also a large planting area in Asia for rape. Among these crops, certain insects and other arthropods are serious agricultural pests, causing significant crops loss if not controlled. Some of them, e.g. moth larvae (Lepidoptera) directly feed on the rhizome and leaves of crops while others mainly feed on nonharvested portions of the plant or suck on plant juices, such as aphids and leafhoppers [18]. Being damaged by these pests, an estimated 18–20% of the annual crop production worldwide is destroyed, estimated at a value of more than $470 billion [19].

When considering the targeted field crops and pest species, using computer vision for pest monitoring is expected to have a domain specific dataset. However, current public datasets for agricultural pest recognition and detection have several limitations: (1) most of them typically cover a small number of samples [20,21], which results in poor generalization, so that the model might not work well on recognizing pests with various attitudes. (2) Many datasets that are target at solving the problem of pest recognition, in which pest objects occupy a large ratio in images [22,23]. However, pests always show to be with tiny sizes in real-life scenes. Besides, most of the images in these datasets contain only one insect pest category, which might be unusual in practical pest images. (3) Some of the datasets collect images in laboratory or non-field environment while using trap devices or from Internet, where these pest images hold a highly simple background, making it difficult to cope with the complexity of practical fields [9,24].

In this paper, we introduce a domain-specific benchmark dataset, called AgriPest, in tiny wild pest detection, providing the researchers and communities with a standard large-scale dataset of practically wild pest images and annotation, as well as standardized evaluation procedures. Different from other public object detection datasets, such as MS COCO [1] and PASCAL VOC [2], which are collected by searching on the Internet, a task-specific image acquisition equipment is designed to build our AgriPest. During this process, we spend over seven years collecting the images due to seasonal and regional difficulty. AgriPest captures 49.7K images of four fields’ crops and 14 species of pests by smartphone in the field environment. All of the images are manually annotated by agricultural experts with up to 264.7K bounding boxes of locating pests. This paper also offers a detailed analysis of AgriPest, where the validation set is split into four types of scenes that are common in practical pest monitoring applications. Benefiting to the practical precision agriculture applications, our AgriPest could provide a large amount of valuable information for precise pest monitoring that could help to reduce crop production loss. Specifically, the current agriculture automation system could deploy a deep learning pest detection method for building effective pest management policy, such as choice and concentration of pesticide, as well as natural enemies controlling and production estimation. We believe our efforts could benefit future precision agriculture and agroecosystems.

The major contributions of this paper lie in three folds:

- To the best of our knowledge, the largest scale domain-specific dataset AgriPest containing more than 49.7K images and 264.7K annotated pests is published for tiny pest detection research. This benchmark will significantly promote the effectiveness and usefulness of applications of new object detection approaches in intelligent agriculture, e.g., crop production forecast.
- AgriPest defines, categories, and establishes a series of detailed and comprehensive domain-specific sub-datasets. Its first category contains two typical challenges: pest detection and pest population counting. Subsequently, it categories four types of the validation subsets of AgriPest dense distribution, sparse distribution, illumination variations, and background clutter, which are common in practical pest monitoring applications.
- Accompanying AgriPest, we build the practical pest monitoring systems that are based on deep learning detectors deployed in the task-specific equipment, in which we give comprehensive performance evaluations of the state-of-the-art deep learning techniques in AgriPest. We believe that AgriPest provides a feasible benchmark dataset and facilitate further research on the pest detection task well. Our dataset and code will be made publicly available.

## 2. Related Work

The emergence of deep learning techniques has led to significantly promising progress in the field of object detection [25], such as SSD [4], Faster R-CNN [5], Feature Pyramid Network (FPN) [6], and other extended variants of these networks [26,27,28,29]. CNN has exhibited superior capacities in learning invariance in multiple object categories from large amounts of training data [23]. It enables suggesting object proposal regions in the detection process and extract more discriminative features than hand-engineered features. The experimental results on the MS COCO [1] and PASCAL VOC [2] dataset show that Faster R-CNN [5] is an effective region-based object detector towards general object detection in the wild with an Average Precision (AP) up to 42.7% with IoU 0.5. In Faster R-CNN, Region-of-Interest (RoI) pooling is used to extract features on a single-scale feature map. However, targeting at small object detection, FPN [6] is the state-of-the-art technique for small object detection over MS COCO dataset with AP up to 56.9% with IoU 0.5. By building up a multi-scale image pyramid, FPN enables a model to detect all of the objects across a large range of scales over both positions and pyramid levels. This property is particularly useful to tiny object detection.

Benefitting from the success of these object detection methods, many applications have been developed in recent years [30,31,32]. Towards pest detection in the wild, deep learning methods might not achieve satisfactory performance, because an excellent object detection application using deep learning techniques usually need to be trained by large enough training dataset. Although there exist a few datasets for solving agricultural issues [33,34], most public large-scale datasets for tiny objects, especially agricultural pest images, cover limited data volume, which causes deep learning methods on pest detection to be restricted [21,22,23]. Besides, a large number of current pest related datasets are collected in the controlled laboratory or non-field environment, which could not satisfy the practical requirements of pest monitoring applications in the field [24]. Moreover, these datasets mainly focus on the pest recognition task, rather than pest detection, so the pest objects occupy a large ratio in images [20]. On the contrary, our proposed AgriPest is built to address practical issues in pest monitoring applications, so all of the images are collected in the wild fields and each pest is annotated with bounding box for detection as well as pest population counting.

## 3. AgriPest Dataset

### 3.1. Taxonomy

**Figure 2:**
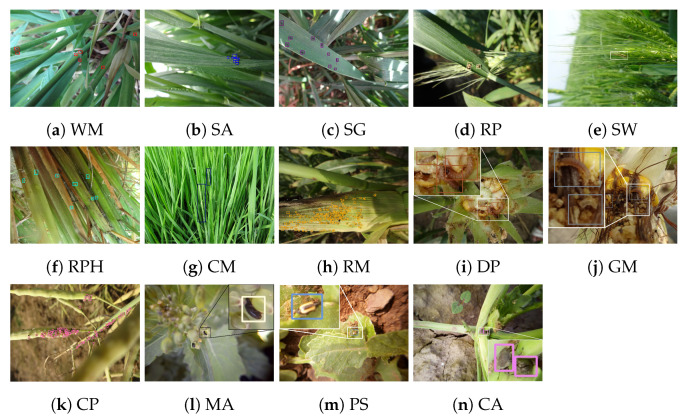
Sample visualization for each pest category in AgriPest. (WM: Wheat mite, SA: Sitobion avenae, SG: Schizaphis graminum, RP: Rhopalosiphum padi, SW: Sticky worm, RPH: Rice planthopper, CM: Cnaphalocrocis medinalis (symptom), RM: Rhopalosiphum maidis, DP: Dichocrocis punctiferalis, GM: Guenee Mythimnaseparata walker, CP: Cruciferae padi, MA: Meligethes aeneus, PS: Phyllostachys striata, CA: Ceuthorrhynchus asper).

IP102 provides its pest taxonomy from 102 pest species [23]. However, among these pest insects, lots of them are not necessary to be prevented in practical agriculture because of their low level for harming fields in certain types of crops. Besides, there are several works that points out rice and wheat are two major crops that are degraded by pests [35]. Therefore, we reform the pest taxonomy of IP102 and focus on pests occuring in four types of crops. Finally, we obtain 14 categories of pests in four super-classes corresponding to four common field crops: wheat, rice, corn, and rape. Within these super-classes, each pest is a subordinate class (also known as sub-class) of a super-class. For example, rice planthopper (RPH) is a sub-class that spoils rice crop, which is one of the super-classes. By this taxonomy system, we build a hierarchical structure of pest categories in AgriPest and the sample of each category is visualized in Figure 2.

### 3.2. Image Acquisition

Current datasets, such as MS COCO, usually collect images using Google or Bing image search. However, most of images on the Internet are not suitable for building a practical pest monitoring application. Besides, the pest monitoring task is novel and specific, ordinary cameras might not be convenient for capturing pests in the root of crops. Thus, there is no proper image acquisition devices for our task. In order to make the captured image reasonable for practical pest occurrence in wild field, we come up with the following requirements: (1) each image must contain at least one type of pest species discussed in Section 3.1. (2) The distance between camera and pest should be various to help the diversity of AgriPest. (3) All of the captured pests need to show their different poses and gestures as they are observed in the real world and overlap is also allowed among these pests.

**Figure 3:**
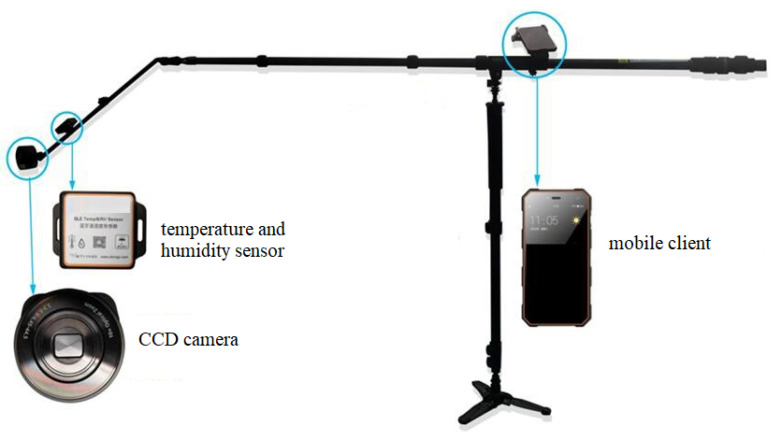
Wild pest image acquisition and pest monitoring equipment.

To meet these requirements, we design a task-specific independent research and development equipment for wild pest image collection whose structure are illustrated in Figure 3. This apparatus is developed with three components in the stand: mobile client, CCD camera, and temperature-humidity sensor. When using this equipment in the field crop, we first adjust the stand height according to the pest locations of the crop, e.g., higher than the crop for wheat, since most pests usually occur in the leaves. Subsequently, we deploy a mobile client and CCD camera in the stand and randomly rotate the hinge of the stand to make the CCD camera cover various viewpoints during image capturing. The parameters of CCD camera are set to 4 mm focal length with an aperture of f/3.3. At the same time, mobile client is connected with CCD camera by wireless network to help users photograph pest images conveniently. In addition, we also adopt a temperature and humidity sensor to record current high-level environment information to help pest annotation process, since some certain pest species might occur under specific environments. Therefore, under our image acquisition, we could capture numerous pest images from the field crop. Finally, we consciously photograph the images in various typical places to improve the dataset diversity in order to balance the distribution of our AgriPest dataset. Furthermore, the candidate images are manually filtered to eliminate those containing few pests. The total number of images captured in AgriPest is 49.7K.

### 3.3. Professional Data Annotation

We invite 20 agricultural experts to annotate these images that are filtered from raw data, who are experienced and knowledgeable in agriculture area, due to our desire to label numerous object instances in 49.7K agricultural images of our large-scale dataset. Specifically, we cooperate with the researchers from Academy of Agricultural Sciences and associate professors in the School of Agriculture and Forestry, which make up of our image annotation team. In order to guarantee the correctness of the annotations, each expert only focuses on pest species of one sup-class so these invited experts are grouped into four groups, each of whose is responsible for annotating the corresponding crop, so it could be ensured that every image is annotated by at least five agricultural experts. In data annotation, the images are first categorized into their sup-classes. The sup-class could be perfectly categorized, because the image source is recorded during collection. Subsequently, the expert groups annotate the pest species and their locations using bounding boxes, respectively. Finally, all of the experts synergistically check the correctness of each labeled instance. The final object instance annotation results follow this criterion: one bounding box and its category could be accepted only when it is agreed by over five experts. The bounding boxes annotations of pests follow the format of Pascal VOC.

### 3.4. Dataset Structure and Splits

For validating the practical application value of AgriPest, we randomly split the whole images into training and validation subsets, which are split at the sub-class level. In total, AgriPest is split into 44,716 training and 4991 validation images for pest detection in the wild task. Besides, we attempt to split these images by keep the similar ratio at different sup-classes, which could ensure the distribution of validation subset is the same as training subset. Table 1 illustrates detailed splits of these two subsets. Note that the sizes of pests would not occupy at most 3% areas over the whole image, because we aim to detect pests of tiny sizes. Furthermore, to investigate various types of scenes in the practical pest detection, we further manually split the validation subset into four types of scenes, including dense distribution, sparse distribution, illumination variations, and background clutter, which are typical scenes in pest monitoring applications. Note that there exist gaps among the four validate subsets, e.g., Wheat Sticky is not in “dense” subset. We explain this phenomenon by various habits of pest species, in which several kinds of pests damage the field without group occurrence, while some other ones usually gather into clique in the field crops. Table 2 illustrates the detailed statistics of these four challenges.

### 3.5. Comparison with Other Datasets

We compare AgriPest with several existing datasets from two aspects, i.e., comparison with generic object detection datasets and comparison with datasets that are related to the task of insect pest recognition or detection to further motivate the construction and usage of our dataset. Table 3 illustrates the comparison.

**Figure 4:**
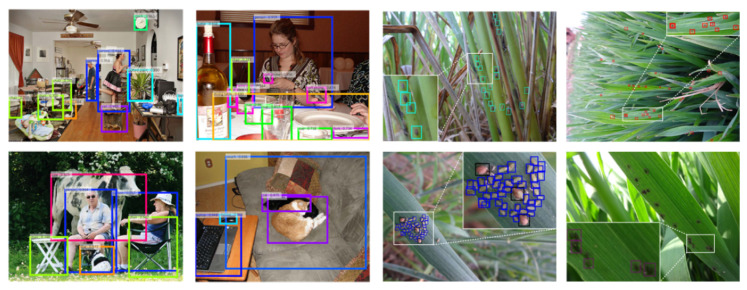
Comparison of PASCAL VOC and our domain-specific AgriPest dataset.

When compared to the PASCAL VOC dataset, which is one of the largest and typical generic object detection datasets, our AgriPest contains over four times more sample images and eight times more annotated objects. In addition, both PASCAL VOC and MS COCO organize lots of common categories of objects in their images so the average size of targeted objects shows to be large (16.76% and 7.74% areas over whole image respectively). However, for in-field tiny pest objects, AgriPest tends to concentrate more their real-life body sizes, in which the pests only occupy average 0.16% area over the whole image in AgriPest that is dozens of times smaller than those in generic object detection datasets, as shown in Figure 4. When compared to traditional generic object detection task that only supports single-depth taxonomy hierarchy and single-scenario in test set, pest monitoring requires a high-level complicated validation metho. Therefore, AgriPest could provide hierarchical categories for pest samples and multi-scenario validation, as shown in Table 3.

**Figure 5:**
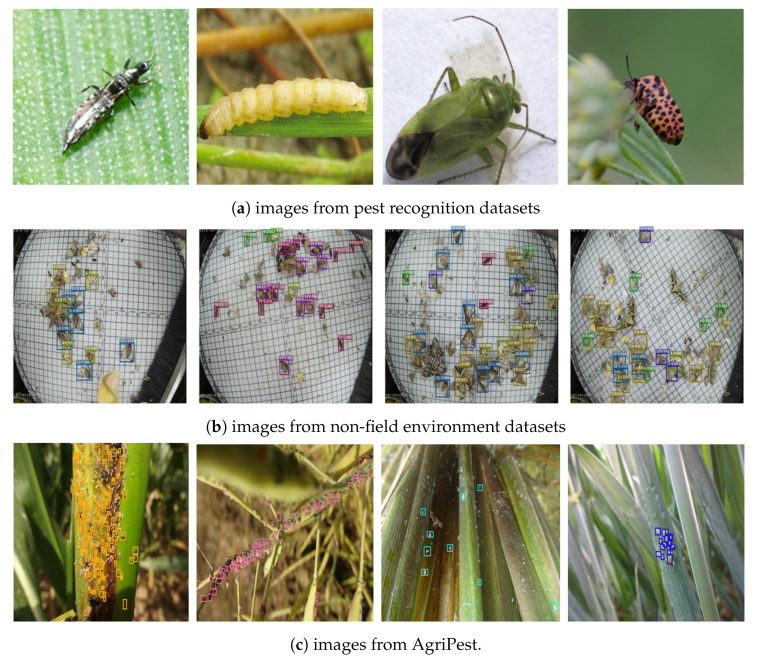
Comparison with other insect pest datasets.

With respect to comparison with some other existing insect pest datasets, our AgriPest could also take great advantages over other datasets for the pest classification task [20,21,22] (Figure 5a), while the insect pests show to be small in AgriPest images. In terms of background, a few current pest detection datasets contain images that are captured from the non-field environment [23] (Figure 5b). Under these limitations, most existing insect pest datasets are difficult to be applied to practical pest monitoring applications. AgriPest targets at tiny pest detection task to meet the requirements of practical applications. Furthermore, AgriPest still cover a larger number of images collected in the wild fields than those current insect pest datasets (Figure 5c).

## 4. Experiments

### 4.1. Experimental Settings

For pest recognition and detection task, the choice of feature is treated as the most significant component. To comprehensively evaluate our AgriPest dataset, we adopt deep learning architectures as benchmarks. For this pest detection task, we select several state-of-the-art methods that are categorized into one-stage architectures and two-stage region-based architectures, including SSD [4], RetinaNet [36], FCOS [37], Faster R-CNN [5], FPN [6], and Cascade R-CNN [38].

We choose VGG16 [39] as CNN backbone for SSD and ResNet-50 [40] the other object detection approaches that are pretrained on ImageNet [13] and then fine-tuned on AgriPest. For fair comparison, the learning algorithms and hyper-parameters are set to be same and all of the models are trained to be optimal. Specifically, a Mini-batch Stochastic Gradient Descent [41] is used as our optimizer with the batch size of 2. The base learning rate is set to 0.01 and linear drop strategy for learning policy is used in our experiments, in which the learning rate drops by 0.1 at 8th and 11th epoch and the total training epoch is 12 referenced by Detectron [42]. The weight decay and momentum parameters are set to 0.0001 and 0.9, respectively. The experiments are implemented using PyTorch and performed on two NVIDIA 1080Ti GPUs with 12 GB memory.

### 4.2. Evaluation Metrics

We employ several comprehensive metrics for evaluation in order to evaluate the performance of CNN models on AgriPest. In our AgriPest, there are two sub-tasks for pest monitoring including pest detection and pest population counting. Firstly, we utilize the Average Precision (AP) with Intersection over Union (IoU) in [0.50:0.05:0.95], AP${}^{0.50}$ and AP${}^{0.75}$ as the pest detection performance evaluation metrics. The IoU is defined as the intersection over the union between predicted box and ground truth. Besides, Precision and Recall are also two major metrics that are employed in our dataset, which describe the false positive reduction and misdetection rate respectively. Secondly, for the pest population counting challenge, we evaluate different models with both the Mean Absolute Error and Mean Squared Error by following the convention of crowd counting task [43]. The MAE and MSE would be averaged among classes. Generally, MAE measures the pest population counting accuracy while MSE measures the robustness of the estimates.

## 5. Results and Discussion

### 5.1. Wild Tiny Pest Detection Results

On the AgriPest dataset, we build some experiments to evaluate the performance of our approach. We select six state-of-the-art object detection methods for comparison, three of which are one-stage architectures (SSD512, RetinaNet, and FCOS), while the other three are two-stage methods (Faster R-CNN, FPN, and Cascade R-CNN).

Table 4 shows the multiclass tiny pest detection performance under these methods. Generally, two-stage architectures could achieve better performance than one-stage methods, outweighing approximately two to four points AP. This could be explained by that most of pests in our AgriPest hold tiny sizes so the coarse-to-fine object detection strategy adopted by region-based methods could lead to more precise pest classification with fine features. This phenomenon seems to be much more pronounced on smaller objects. For example, pest CP that holds 0.006% size gets over 10 points AP improvement between these two types of methods. Among these approaches, SSD512 performs poorly on most categories of pests. This indicates that, when the image is scaled to be small (512 × 512 resolution), the features of targeted tiny pests might be hard to extract and current state-of-the-art methods still could not satisfy real-world applications.

In addition, we illustrate the detection results using AP${}^{0.50}$, AP${}^{0.75}$, and AP${}^{\left[0.50:0.05:0.95\right]}$ as metrics in Table 5 and Table 6. As it could be observed, most of methods could obtain satisfied performance on IoU 0.5, but obtain a significant decrease when a higher IoU is the set AP threshold. Thus, existing object detection methods might not work well on highly precise pest localization, because lots of ground truths are too small to be localized. Overall, these detection results demonstrate that AgriPest exhibits high difficulty on wild tiny pest detection as well as its research value.

### 5.2. Precision-Recall Analysis

**Figure 6:**
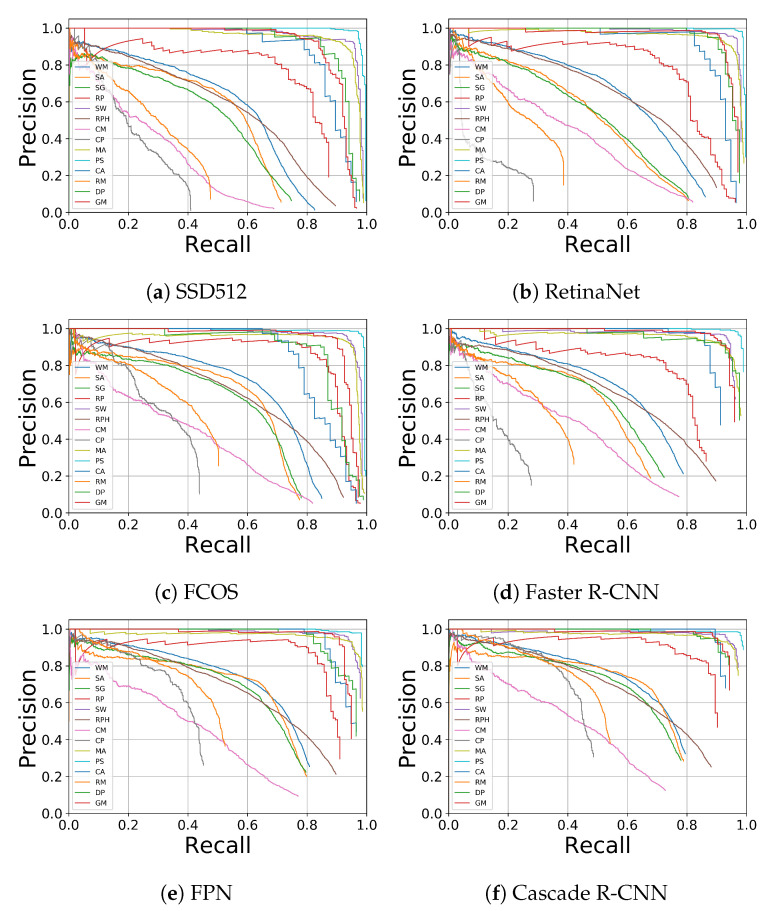
Comparison with other insect pest datasets.

We evaluate the Precision-Recall (PR) by comparing the six object detection methods in AgriPest shown in Figure 6 in order to further analyze the detailed detection results. It is obvious that these methods could obtain the satisfied performance for most of pest categories especially for those with relatively large sizes, such as pest SW, DP, and GM. However, for a few certain classes, such as RM and CP, existing methods might not work well on misdetection reduction (low recall). Furthermore, the precision keeps dropping dramatically with the slight improvement of recall, which indicates that false positive reduction might also not be well performed. This could be attributed to two reasons. Firstly, for these ‘hard’ categories, there are a large number of pests that are densely distributed in each image (around 60 pests per image for RM and 50 for CP), leading to poor recall performance, which is also evidence for low AP from Table 2. Secondly, the training samples for RM and CP are insufficient in AgriPest, in which there are 189 and 193 images for training, respectively. In this case, models may not effectively learn the highly discriminative features for these pests from their background context.

### 5.3. Scene Analysis

Table 7 illustrates the detection results in dense distribution, sparse distribution, illumination variations, and background clutter using Cascade R-CNN [38] as a pest detector in order to evaluate the influence of various scenes for wild pest detection. The results show that pests sparsely distributed in images are the easiest to be detected, with more than 70% AP being obtained for most of pest species, while dense distribution is the most difficult challenge for wild tiny pest detection. This is in line with the conclusion that most of object detection approaches could not detect well on pest RM and CP, which usually occur with dense cliques in the field. On the contrary, for the pest species that do not gather together, the detector could perform well on detection, even when the background is cluttered. Apart from the distribution influence, illumination variation is also the unavoidable challenges in practical pest monitoring.

### 5.4. Pest Population Counting Results

In AgriPest, pest population counting is another task for practical pest monitoring applications, because precise population estimation is important in assessing crop damage degree and pest severity. Table 8 presents the average Mean Absolute Error (MAE) and Mean Square Error (MSE) while using six object detection methods for pest population counting. As it can be seen, Faster R-CNN achieve the best results in both MAE and MSE. Besides, two-stage approaches dramatically outperform one-stage approaches in this task. Thus, for tiny pest counting, region-based methods could precisely maintain the correctness of detected pest population.

**Figure 7:**
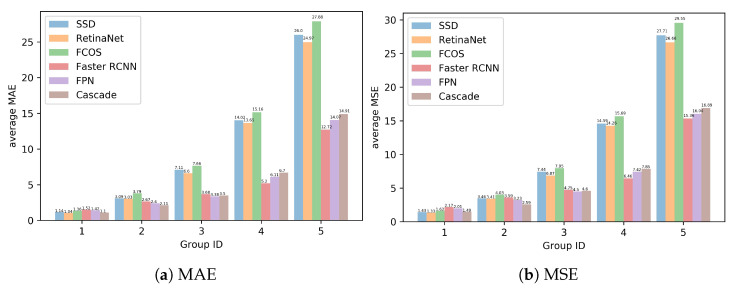
Average MAE and MSE for pest population counting with six state-of-the-art methods.

In Figure 7, we compare these object detection methods in greater details. We evenly group the test images of each classes into 5 groups according to pest population in an increasing order. From this figure, it can be seen that, in the group 1 and 2, where the images contain a few pests, the six methods seem to show the similar population counting performance, which indicates that these approaches might not incorrectly detect pests. With the number of pests increasing, the errors, including absolute error and squared error, start boosting and the difference between two types of methods also becomes larger. Therefore, it is verified that two-stage methods perform more accurately and robustly to a large variance of pest number as well as density.

### 5.5. Limitations and Future Work

**Figure 8:**
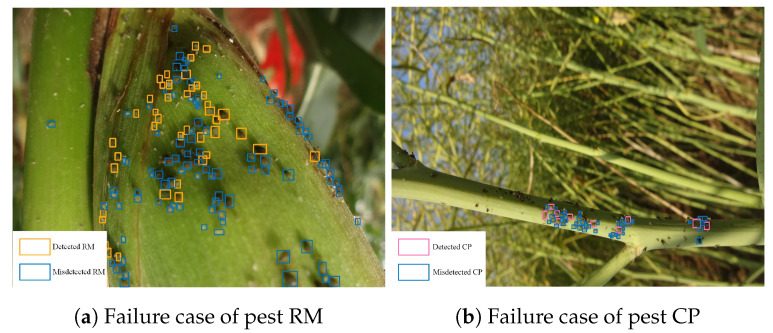
Detection result visualization for pest RM and CP.

Despite that we implement some state-of-the-art object detection approaches with good performance in AgriPest, there are two limitations for future study. Firstly, the problem of unbalanced data structure has not been well solved. Specifically, pest RM and CP are two difficult pest categories in wild pest detection, because AgriPest does not contain sufficient data for model to learn them while they usually occur in cliques with tiny sizes in images, as visualized in Figure 8. Secondly, employing existing generic object detection approaches in our wild tiny pest detection task is not a qualified solution. Future work will focus on covering a larger number of categories and it develops a novel domain-specific algorithm for this task.

## 6. Conclusions

In this work, we collect a domain-specific benchmark dataset, named AgriPest, towards large-scale tiny pest detection in the wild. Our dataset covers 49.7K images and 264.7K annotated pest objects of 14 common pest species. When compared with other insect pest dataset, AgriPest targets at wild tiny pest detection in practical science. In addition, the validation images are split into four challenges that are common in practical pest monitoring applications. The images in AgriPest are collected by our designed task-specific equipment that is also deployed in practical pest monitoring application in the field. We implement and evaluate some state-of-the-art generic object detection methods in AgriPest. The experimental results demonstrate the difficulty and particularity of our AgriPest. We believe this work will help to advance future research on wild pest detection task and practical precision agriculture applications. 

## Figures and Tables

**Table 1:** Statistics on Two Subsets for AgriPest with training subset and validation subset. ‘size (%)’ indicates the average areas of pests over the whole images. For each class, the number of images (containing at least one insect the class), the number of objects and the average number of objects per image are shown in this table. Note that, because single image may contain objects of several classes, the totals in the ‘#images’ columns are not simply the sum of the corresponding columns.

Field Crop	Pest Name	Size(%)	Training		Validation		All	
			#Images	#Objects	#Images	#Objects	#Images	#Objects
wheat	WM	0.089	11,505	54,423	1278	6095	12,783	60,518
	SA	0.086	5230	26,385	588	2844	5818	29,229
	SG	0.075	5997	34,262	656	3819	6653	38,081
	RP	0.100	697	1634	72	133	769	1767
	SW	1.512	2901	2980	303	320	3204	3300
rice	RPH	0.143	9572	72,027	1074	8268	10,646	80,295
	CM	0.407	2215	14,352	247	1580	2462	15,932
corn	RM	0.042	189	11,865	36	2325	225	14,190
	DP	2.930	529	576	70	84	599	660
	GM	2.941	1565	1699	155	174	1720	1873
rape	CP	0.060	193	9328	15	699	208	10,027
	MA	0.061	3668	5035	404	570	4072	5605
	PS	0.391	2368	2421	276	281	2644	2702
	CA	0.325	388	492	44	57	432	549
total			44,716	237,479	4991	27,249	49,707	264,728

**Table 2:** Statistic on validation subset of AgriPest split in four typical scenes. Note that single image may contain more than one type of scene, the totals shown in the ‘#images’ columns are not simply the sum of the corresponding columns.

Field Crop	Pest Name	Dense		Sparse		Illumination		Background Clutter	
		#Images	#Objects	#Images	#Objects	#Images	#Objects	#Images	#Objects
wheat	WM	178	1745	1064	4205	214	760	56	324
	SA	236	2036	241	632	211	935	31	121
	SG	210	2296	310	1199	209	1014	69	700
	RP	17	46	55	87	25	49	15	27
	SW	-	-	303	320	105	109	236	253
rice	RPH	116	1918	876	8925	77	482	1052	8095
	CM	74	654	99	504	21	145	63	355
corn	RM	36	2325	-	-	6	261	28	2010
	DP	-	-	70	84	-	-	66	80
	GM	-	-	155	174	21	23	134	151
rape	CP	11	663	3	24	2	172	4	24
	MA	-	-	375	502	1	2	37	101
	PS	-	-	270	274	-	-	21	24
	CA	-	-	18	31	-	-	26	26
total		786	11,683	3743	16,961	835	3952	1811	12,291

**Table 3:** Comparison with Other datasets. ‘Det.’ and ‘Rec.’ indicate the object detection and image recognition task that datasets focus on.

Dataset	Task	#Images	#Objects	Size(%)	Collection Environment	Hierarchical Categories	Multi-Scenario Validation
PASCAL VOC [2]	Det.	33K	79K	16.76	-	-	-
MS COCO [1]	Det.	123K	896K	7.74	-	-	-
Xie et al. [22]	Rec.	4.5K	-	-	in-field	✓	-
Alfarisy et al. [21]	Rec.	4.5K	-	-	in-field	-	-
Liu et al. [20]	Rec.	5.1K	-	-	in-field	-	-
IP102 [23]	Rec.	75K	-	-	in-field	-	-
Ding et al. [9]	Det.	0.2K	4.4K	-	non-field	-	-
MPD2018 [24]	Det.	88K	580K	0.26	non-field	-	-
AgriPest	Det.	49K	264K	0.16	in-field	✓	✓

**Table 4:** Average Precision (AP) for Multiclass Pest Detection Results with Intersection over Union (IoU) 0.5. The pest categories are summarized into three groups according to their size proportion in image. **Large:** SW, DP, GM (∼3%), **Middle:** CM, PS, CA (∼0.4%), **Small:** WM, SA, SG, RP, RPH, RM, CP, MA (<0.1%).

Method	Wheat					Rice		Corn			Rape				
	WM	SA	SG	RP	SW	RPH	CM	RM	DP	GM	CP	MA	PS	CA	Mean
SSD512 [4]	54.09	48.69	45.74	71.84	89.66	54.68	28.36	29.75	88.52	87.63	24.42	89.02	90.84	84.04	63.38
RetinaNet [36]	59.60	47.79	48.17	76.15	90.44	60.45	39.41	25.76	90.32	89.82	14.46	89.05	90.88	88.00	65.03
FCOS [37]	62.83	55.98	54.21	83.60	90.15	62.40	38.85	39.18	85.15	89.81	33.51	88.35	90.55	81.66	66.22
Faster R-CNN [5]	57.67	47.68	51.59	72.64	89.78	60.15	40.72	32.83	89.00	90.15	18.67	88.49	90.87	87.86	65.58
FPN [6]	63.05	58.19	57.58	81.92	90.19	62.13	40.38	44.50	88.76	89.97	37.92	88.81	90.80	88.11	70.20
Cascade R-CNN [38]	60.99	58.95	58.32	82.61	89.85	62.38	41.44	46.27	90.09	89.96	40.75	89.14	90.58	90.26	70.83

**Table 5:** AP${}^{0.75}$ performance of pest detection methods.

Method	Wheat					Rice		Corn			Rape				
	WM	SA	SG	RP	SW	RPH	CM	RM	DP	GM	CP	MA	PS	CA	Mean
SSD512 [4]	17.14	16.55	9.08	35.32	41.56	16.28	4.32	5.06	45.21	46.12	3.14	44.26	41.21	41.97	26.23
RetinaNet [36]	19.23	17.89	10.84	38.15	44.88	19.01	6.21	7.51	47.32	48.24	6.28	47.23	44.54	48.25	28.97
FCOS [37]	19.21	18.01	10.62	38.03	42.98	21.06	6.02	7.15	46.88	48.15	5.04	45.56	42.78	50.58	28.72
Faster R-CNN [5]	18.04	17.21	10.13	36.02	42.78	17.54	5.62	6.77	46.33	47.57	4.41	45.69	42.47	45.54	27.58
FPN [6]	19.98	20.01	12.54	38.26	42.50	22.17	7.18	9.14	47.95	48.76	7.05	47.03	44.51	51.65	29.91
Cascade R-CNN [38]	20.85	23.84	14.01	41.16	44.41	23.45	11.06	11.97	49.63	50.10	12.12	49.87	45.82	53.77	32.29

**Table 6:** AP${}^{\left[0.50:0.05:0.95\right]}$ performance of pest detection methods.

Method	Wheat					Rice		Corn			Rape				
	WM	SA	SG	RP	SW	RPH	CM	RM	DP	GM	CP	MA	PS	CA	Mean
SSD512 [4]	20.81	21.12	13.43	39.45	43.12	23.47	7.94	10.07	49.03	49.47	8.31	48.15	45.41	52.12	30.85
RetinaNet [36]	24.16	24.84	15.97	41.81	45.62	26.73	11.02	11.96	52.15	53.03	10.56	50.32	47.28	52.85	33.45
FCOS [37]	25.62	26.13	14.05	40.76	44.51	26.03	11.56	12.04	51.43	52.78	11.04	49.43	46.78	53.20	33.24
Faster R-CNN [5]	22.84	24.89	13.67	40.45	44.13	24.72	11.15	10.83	52.17	52.54	10.01	47.46	45.26	51.52	32.26
FPN [6]	25.12	27.78	17.71	42.86	47.54	28.10	13.23	14.02	55.16	54.36	12.84	51.73	47.15	55.34	35.21
Cascade R-CNN [38]	27.94	29.63	20.15	44.07	48.96	30.01	15.14	15.63	55.56	54.89	14.41	51.87	47.41	55.89	36.54

**Table 7:** Detection results on four challenges (AP with IoU 0.5). Sparse distribution, illumination variations, and background clutter. We use Cascade R-CNN [38] as the pest detector.

Field Crop	Pest Name	Dense	Sparse	Illumination	Clutter
wheat	WM	35.87	74.71	48.54	60.48
	SA	34.12	72.47	47.98	58.66
	SG	33.96	72.14	47.67	58.15
	RP	62.36	83.84	67.54	82.17
	SW	-	-	89.77	90.21
rice	RPH	38.42	75.72	50.51	62.34
	CM	15.22	51.46	34.15	41.05
corn	RM	19.68	61.26	-	-
	DP	-	-	89.62	91.05
	GM	-	-	88.49	90.23
rape	CP	14.58	56.28	32.74	43.19
	MA	-	-	87.48	89.67
	PS	-	-	89.14	90.88
	CA	-	-	88.85	90.92

**Table 8:** Average MAE and MSE on pest population counting performance.

Method	MAE(avg)	MSE(avg)
SSD512 [4]	9.23	13.28
RetinaNet [36]	8.79	12.76
FCOS [37]	10.09	14.25
Faster R-CNN [5]	4.46	7.41
FPN [6]	4.75	7.66
Cascade R-CNN [38]	4.94	7.97

## Data Availability

Our dataset and code will be made publicly available at https://github.com/liuliu66/AgriPest.
